# Development of “Quadrello di Ovino”, a Novel Fresh Ewe’s Cheese

**DOI:** 10.3390/foods11010025

**Published:** 2021-12-23

**Authors:** Giuliana Garofalo, Gabriele Busetta, Giuseppe Maniaci, Maria Teresa Sardina, Baldassare Portolano, Natale Badalamenti, Antonella Maggio, Maurizio Bruno, Raimondo Gaglio, Luca Settanni

**Affiliations:** 1 Dipartimento Scienze Agrarie, Alimentari e Forestali, Università degli Studi di Palermo, Viale delle Scienze, Ed. 5, 90128 Palermo, Italy; giuliana.garofalo01@unipa.it (G.G.); gabriele.busetta@unipa.it (G.B.); giuseppe.maniaci@unipa.it (G.M.); mariateresa.sardina@unipa.it (M.T.S.); baldassare.portolano@unipa.it (B.P.); raimondo.gaglio@unipa.it (R.G.); 2 Dipartimento di Scienze e Tecnologie Biologiche Chimiche e Farmaceutiche, Università degli Studi di Palermo, Viale delle Scienze, Ed. 17, 90128 Palermo, Italy; natale.badalamenti@unipa.it (N.B.); antonella.maggio@unipa.it (A.M.); maurizio.bruno@unipa.it (M.B.)

**Keywords:** ewe’s milk, fresh cheese, lactic acid bacteria, physicochemical parameters, starter cultures

## Abstract

This work was performed to produce a new soft ewe’s milk cheese, namely “Quadrello di ovino” (QdO) cheese, to enlarge ewe’s dairy product portfolio of South Italy, barely limited to Pecorino cheese typology. Cheese making was performed applying the technology for “Crescenza” cheese typology with some modifications. In particular, pasteurized ewes’ milk was inoculated with two commercial starter formulations (SF1 and SF2) of *Streptococcus thermophilus* to obtain two different productions (QdO-P1 and QdO-P2, respectively). Plate counts demonstrated the ability of both starter formulations to drive the fermentation process, since *S. thermophilus* counts reached 10^9^ CFU/g in both productions. Generally, the two starter formulations did not affect the chemical composition of QdO cheeses that contained, on average, 64.08% dry matter of which approximately 54.99% were fats and 36.39% proteins. Among chemical parameters, significant differences were registered for secondary lipid oxidation state (significantly lower for QdO-P2), fatty acids and volatile organic compounds (VOCs). However, the differences registered among cheese VOCs from were not perceived by the panelists who recognized both cheese productions highly similar, although QdO-P2 cheeses were mostly appreciated by the judges. This study allowed to produce a novel fresh ovine cheese with specific chemical and sensorial characteristics well appreciated by consumers.

## 1. Introduction

The great diversity of cheeses registered worldwide depends on several factors, mainly type of milk, technology applied, starter and secondary cultures inoculated and conditions of ripening [1]. Strictly considering the microbiological aspects, all cheeses are subjected to an internal bacterial transformation, mainly operated by lactic acid bacteria (LAB). Dairy LAB act in distinct phases of cheese production: those involved in the acidification of the curd are known are starter LAB, while the species responsible for the biochemical changes occurring during ripening are indicated as non-starter LAB [2]. Furthermore, during ripening a different spatial distribution of LAB can be found among cheese profile, from under rind to core (central part) [3].

Italy and France are probably the countries with the highest world’s cheese diversity. In particular, Italy is the world’s country with the highest number of food products that enjoy a recognition of quality status. Among these, 56 different cheeses are indicated as protected designation of origin (PDO), protected geographical indication (PGI) or traditional specialty guaranteed (TSG) products [4]. These recognitions are very important for the valorisation of dairy products, especially cheeses that acquired a bad reputation in terms of healthy properties due to their general high fat content [5]. Among cheese products, those mostly affected by this negative image are those processed from ewe’s milk that is particularly rich in fats [6]. 

Besides traditional cheeses, new types of cheeses are being produced for several reasons such as improving the functional properties, to face allergies, to encounter the request of vegetarians, to respond to new lifestyles, to increase economic revenues and also for religious motivations [7,8,9]. In this framework, the milk mostly used for novel cheese productions is cow’s milk. Up to date, innovation in cheese production included the addition of fruit and vegetable by-products to obtain functional cheeses [10,11], the adjunct of natural food colorants to produce more attractive colored products [12], the inclusion of cereals as prebiotics to enhance the development of probiotic bacteria at intestinal level [13], even the incorporation of ripened cheese in novel fresh cheeses to valorize cheese surpluses [14]. However, there is also the need to valorize some milk productions generally addressed to a few specific productions.

Regarding the economic implications of developing new cheeses, in southern Italy there is a strong need to valorize ewe’s milk productions. The two main Italian islands, Sicily and Sardinia, represent the regions where sheep milk is produced at consistent levels [15]. In order to contribute to the rural development and to impact positively on the local regional economy, Sicilian research institutes and cheese producers are collaborating to offer new dairy products with the aim of limiting the land abandonment phenomenon, particularly common in the internal hilly areas [16]. Generally, Italian hard cheeses processed from ewe’s milk are referred to as “Pecorino” cheeses [17]. This cheese typology is often characterized by a strong aromatic flavour [18]. Due to the characteristic aroma, ripened Pecorino cheeses are not appreciated by several people, especially those who are sensitive to strong smells. “Pecorino” is actually an adjective that comes from “pecora”, the Italian translation of ewe, but in common thinking, all cheeses processed from ewe’s milk are identified as cheeses with an aggressive smell, even those that do not undergo a long ripening period. Thus, the major challenge to innovate ewe’s milk cheese production is the manufacture of new products welcomed by consumers. Another dairy segment for which the production of new products is urgent is buffalo’s milk transformation. To this purpose, buffalo’s milk has been used to process Stracchino cheese, a soft cheese belonging to Crescenza typology [19], that was well appreciated by judges [20]. 

The main objective of this work was to produce a new soft ewe’s milk cheese applying the technology of Crescenza cheese typology by means of commercial thermophilic LAB. The final cheeses were characterized for their microbiological and physicochemical traits, volatile organic compounds (VOCs) profile as well as sensory features.

## 2. Materials and Methods

### 2.1. Milk and Starter Culture

Raw ewe’s whole milk was obtained from several farms and transported daily by a temperature-controlled road tanker to the dairy factory “Il Cacio Siciliano” located in Belmonte Mezzagno (Italy). Bulk milk (500 L) was kept refrigerated (4–6 °C) under stirring until cheese making. Pasteurization occurred at 71 °C for 10 s in a PS15351 COMAT pasteurizer (CO.MAT. s.r.l., Bellizzi, Italy). After heat treatment, the milk was cooled at 38 °C to perform starter inoculation. The characteristics of pasteurized milk (average data of the bulks used in this study) were: pH 6.60 ± 0.03; somatic cell count 3.12 ± 0.13 Log, fat 5.86 ± 0.25%; protein 4.99 ± 0.15%; casein 3.87± 0.31%; and lactose 3.81 ± 0.35%.

Two different freeze-dried starter formulations CR/A (SF1) and LYOBAC-D CRM (SF2) were purchased from microMilk s.r.l. (Cremosano, Italy) and Alce International s.r.l. (Quistello, Italy), respectively. Both commercial starter cultures were prepared from defined strains of *S. thermophilus* (SF1: strains CR/A1 and CR/A2; SF2: strains CRM4 and CRM6). Freeze-dried cells (5 Units) were re-activated for 10 min in 2 L of pasteurized ewe’s milk, kept under manual agitation, to prepare starter inoculums. 

### 2.2. “Quadrello di Ovino” Cheese Making

**Figure 1:**
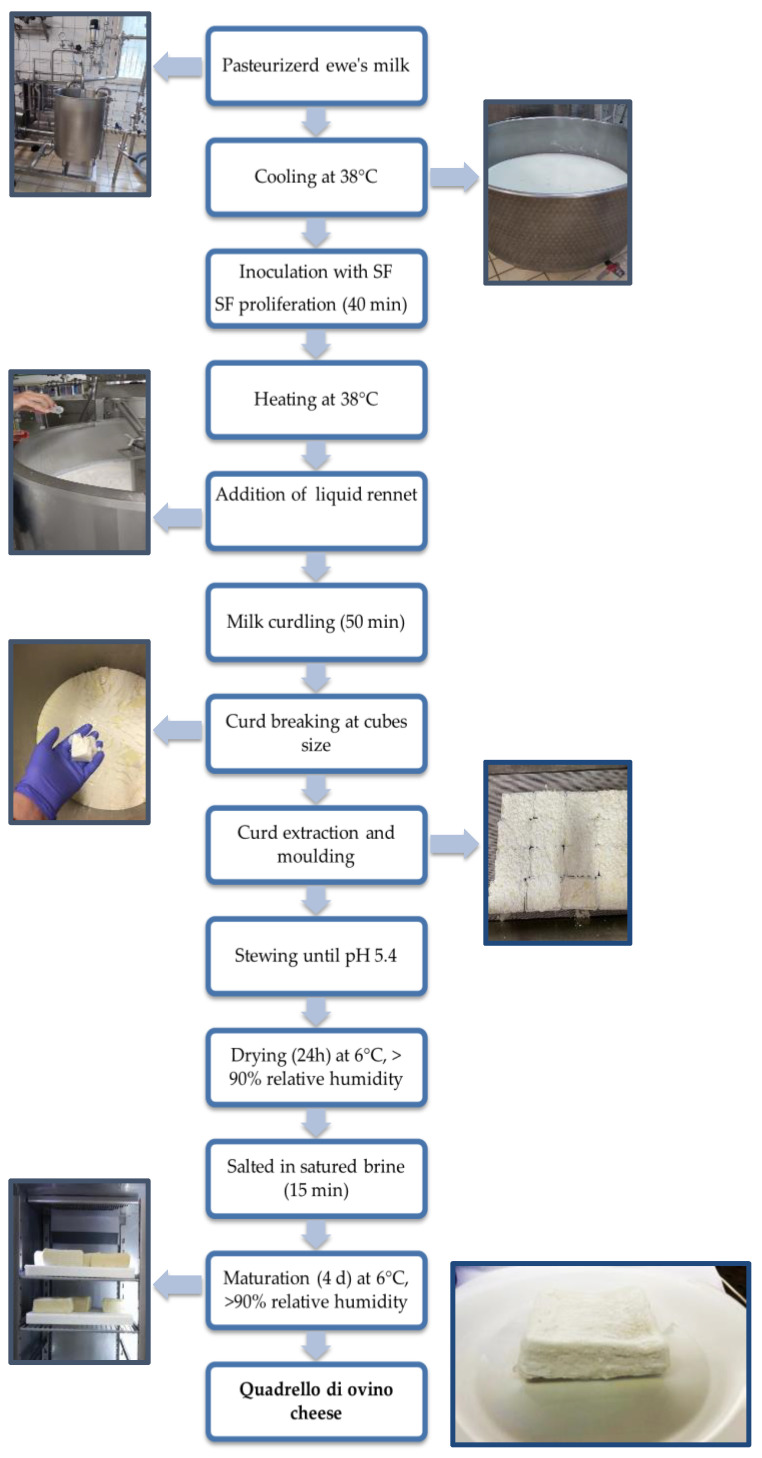
Flow diagram set up for this study to produce “Quadrello di ovino” cheese. Abbreviations: SF, starter formulations.

The novel cheese object of study, “Quadrello di ovino” (QdO) cheese, owes its name to the little square shape given to this sheep milk cheese. QdO production (Figure 1) was performed by modification of the classical flowsheet of “Crescenza” cheese making. The two distinct productions carried out with SF1 and SF2 were indicated as QdO-P1 and QdO-P2.

Bulk milk was transferred to a stainless-steel vat and, after starter preparation inoculation, subjected to a gentle agitation (20 rpm) for 5 min, then left to rest for 40 min. The milk was re-heated by direct steam injection to bring the temperature back to 38 °C and added with 60 mL of Naturen Premium 225 liquid rennet (Chr. Hansen’s Laboratory, Parma, Italy) characterized by a strength of 1:22,500. After about 20 min, the coagulum was first subjected to a coarse cut performed by a stainless-steel rod and, after additional 50 min, the cut was concluded with a cheese harp containing vertical and horizontal wires to obtain cubes of about 3 cm × 3 cm × 3 cm. The curd was transferred into cuboid moulds (10 cm × 10 cm × 7 cm) and stewed under steam at 45 °C for 30 min, turned upside down stewed for other 30 min and then left acidifying until pH 5.4. The cheeses were transferred into a cold chamber [6 °C, relative humidity (RH) > 90%] for 24 h before being immersed into a saturated brine for 15 min and then stored at 6 °C, RH > 90% covered by plastic sheets for 4 d. Finally, the cheeses were packed into plastic boxes and sealed with a transparent film. 

The day after, cheese production was performed with the second starter inoculum and both productions were repeated three times in three consecutive months. Samples of pasteurized milk, inoculated milk, curd and cheese, were collected from each cheese making and subjected to several analyses. 

### 2.3. Microbiological Analyses

Milk samples (1 mL) were serially diluted (1:10) in Ringer’s solution (Sigma-Aldrich, Milan, Italy). Curd and cheese samples (15 g) were homogenized with 2% (*w*/*v*) sodium citrate solution (135 mL) by means of the stomacher Bag-Mixer 400 (Interscience, Saint Nom, France) for 2 min at the maximum speed (blending power—4). Curd and cheese homogenates were then subjected to the decimal serial dilution as reported above in Ringer’s solution.

Cell suspensions of pasteurized milk samples were analysed by plate count for the enumeration of the total mesophilic microorganisms (TMM), LAB, *Pseudomonas* spp., coagulase positive staphylococci (CPS), members of Enterobacteriaceae family, *Escherichia coli*, *Listeria monocytogenes* and *Salmonella* spp. TMM were grown on Skim milk agar ISO 6610 incubated aerobically at 30 °C for 72 h. de Man–Rogosa–Sharpe (MRS) agar and Medium 17 (M17) were used for the growth of rod- and coccus-shaped LAB, respectively, after 48 h incubation at 30 °C for mesophilic groups and at 44 °C for thermophilic groups. *Pseudomonas* species were investigated on *Pseudomonas* agar base (PAB) supplemented with Cephaloridine–Fucidin–Cetrimide (CFC), incubated aerobically at 25 °C for 48 h. Members of Enterobacteriaceae family were detected on violet red bile glucose agar (VRBGA), after incubation at 37 °C for 24 h. *L. monocytogenes* were cultivated on *Listeria* selective agar base (LSAB) added with SR0140E supplement, incubated at 37 °C for 48 h. Hektoen enteric agar (HEA), incubated at 37 °C for 24 h, was specifically used for enteric Gram-negative bacteria (*Salmonella* spp. and *E. coli*), while Baird Parker (BP) agar with rabbit plasma fibrinogen supplement, incubated in the same conditions, for CPS.

Cell suspensions of inoculated milk, curds and cheeses were analysed for TMM and thermophilic coccus-shaped LAB on M17 agar as reported above. All media and chemicals were purchased from Oxoid (Milan, Italy). Plates counts were performed in triplicate.

### 2.4. Physicochemical Analyses

#### 2.4.1. Physical Determination

Color of internal surfaces of the cheeses was assessed by a Minolta Chroma Meter CR300 (Minolta, Osaka, Japan) using the illuminant C; measurements of lightness (L*, from 0 = black, to 100 = white), redness (a*, from red = +a, to green = -a) and yellowness (b*, from yellow = +b, to blue = −b) were performed according to the CIE L* a* b* system [21].

Cheese hardness was evaluated by the maximum resistance to compression (compressive stress, N/mm^2^) of samples (2 cm × 2 cm × 2 cm) kept at room temperature (22 °C). This parameter was measured as index of cheese hardness with an Instron 5564 tester (Instron Corp., Trezzano sul Naviglio, Milan, Italy). 

#### 2.4.2. Content of Basic Ingredients and Antioxidant Activity

Cheese samples were freeze-dried by a SCANVAC Coolsafe 55–9 (Labogene Aps, Lynge Denmark) and analysed for content of basic ingredients as reported by Bonanno et al. [22]. Briefly, dry matter (DM) was determined after drying in an oven at 105 °C until constant weight, fat content through the extraction of fatty substances with petroleum ether and diethyl, after acid hydrolysis, protein content using the Kjeldahl method (N × 6.38), ash content through calcitation at 550 °C, and NaCl content through the precipitation of chlorides with the addition of silver nitrate and subsequent titration with ammonium sulphocyanide. The determination of soluble nitrogen was carried out by treatment with a sodium citrate solution and subsequent precipitation of the proteins at pH 4.6 and was determined on aqueous filtrate using the Kjeldahl method, the proteolysis index (PI) was calculated as the percentage ratio between NPN and total nitrogen (TN) [23]. 

The oxidation status of fat was assessed in the freeze-dried cheese samples by determining the peroxide value (POV, mEq O_2_/kg fat) as an index of primary lipid oxidation [24], in screw-cap Pyrex culture tube, 20 mg of fat are dissolved with 9.8 mL of a chloroform-methanol solution (70:30) and added with 50 µL of iron chloride and 50 µL of ammonium thiocyanate, after 5 min of incubation it is read the absorbance at 500 nm using an Hach DR3900 spectrophotometer (Hach Company, Ames, Iowa, USA). The products of secondary lipid oxidation were determined as thiobarbituric acid reactive substances (TBARS), expressed as μg malonylaldehyde (MDA)/kg DM, as reported by Bonanno et al. [22]. In brief, phosphate buffer aqueous solution (pH 7.0) (8 mL) was added to cheese (2 g) in a 25-mL Sovirel tube, and the mixture was homogenized using an Art-Miccra D-8 high-speed homogenizer (Moderne Labortechnik, Heitersheim, Germany). A 30% (*v*/*v*) trichloroacetic acid aqueous solution (2 mL) was then added and the sample was mixed in a vortex mixer for a few seconds, then filtered through Whatman No. 1 filter paper. A 0.02 M aqueous solution (5 mL) of thiobarbituric acid was added to 5 mL filtrate. The solution was placed in a hot water bath (90 °C) for 20 min then refrigerated. The absorbance of the supernatant was read at 530 nm using a Hach DR3900 spectrophotometer (Hach Company), after centrifugation at 4500 rpm for 5 min. TBARS were quantitatively determined, using 1,1,3,3-tetramethoxypropane solutions at concentrations ranging from 0.016 to 0.165 μg/mL for the calibration curve (R2 = 0.99). Physicochemical determinations were performed in triplicate.

### 2.5. Determination of Cheese Fatty Acids

Fatty acids (FAs) were directly methylated in screw-cap Pyrex culture tube on lyophilized samples (500 mg) with the addition of 0.7 mL of 10 N KOH in water, 5.3 mL of MeOH and 0.58 mL of 24 N H_2_SO_4_ in water [25]. Methyl ester of C23:0 (Sigma-Aldrich, Milan, Italy) was used as internal standard (0.5 mg/g freeze-dried sample) for the FA quantification. Fatty acid methyl esters (FAME) were recovered in hexane (1.5 mL). Each sample (1 μL) was injected by autosampler into an Agilent 7000C GC system, fitted with a fused silica DB-5MS capillary column (30 m × 0.25 mm i.d.; 0.25 μm film thickness) (Santa Clara, CA, USA) coupled to an Agilent triple quadrupole Mass Selective Detector MSD 5973; ionization voltage 70 eV; electron multiplier energy 2000 V; transfer line temperature, 270 °C. Solvent Delay: 0 min. Helium was the carrier gas (1 mL/min). The oven temperature was held at 70 °C for 1 min, increased of 5 °C/min to 100 °C, held for 2 min, increased of 10 °C/min to 175 °C, held for 40 min and finally increased at 5 °C/min to 225 °C, held for 45 min. The identification of each FAs was performed as described by Alabiso et al. [26]: the individual FAs were identified by comparing their retention times with those of a standard FAs (FAME mix C4-C24, CRM47885 Supelco, and tricosanoic acid conjugated methyl ester, Sigma-Aldrich). The health-promoting index (HPI) was calculated as reported by Ashkezary et al. [27]: (n-3 PUFA + n-6 PUFA + MUFA)/(C12:0 + (4 × C14:0) + C16:0).

### 2.6. Volatile Organic Compound Determination

The method proposed by Tunick and co-workers [28] was followed for the determination of the volatile compounds of cheeses. All cheese samples were stored in the freezer at −15 °C and subsequently defrosted at 20 °C in a fridge. Five grams of each individual sample were grated and placed into a 20 mL vial (75.5 × 22.5 mm) (Supelco, Bellefonte, PA, USA). Each sample was added with an internal standard solution (0.1 mL). The internal standard solution was ethyl benzoate (Sigma Aldrich, St. Louis, MO, USA) at 5 ppm. The most suitable internal standard was chosen to quantify each analyte, depending on the best linearity and precision parameters found. A fiber assembly was evaluated and used: 50/30 µm divinylbenzene (DVB)/carbowax (CAR)/polydimethylsiloxane (PDMS) (Supelco^®^, Bellefonte, PA, USA). The samples were equilibrated at 40 °C for 30 min. The SPME fiber was exposed to the cheeses for 30 min in the headspace of the sample kept at 40 °C. The flavour compounds were desorbed for 10 min from the fiber to the column through a splitless injector at 250 °C. Before use, fiber was conditioned and cleaned at 270 °C for 30 min, following instructions from Supelco^®^. All samples were prepared in triplicates in standard 20 mL-volume headspace vials. 

Quantification of volatile compounds was performed using an Agilent 7000C GC system, fitted with a fused silica DB-5MS capillary column (30 m × 0.25 mm i.d.; 0.25 μm film thickness) (Santa Clara, CA, USA) coupled to an Agilent triple quadrupole Mass Selective Detector MSD 5973; ionization voltage 70 eV; electron multiplier energy 2000 V; transfer line temperature, 270 °C. Solvent delay: 0 min. Helium was the carrier gas (1 mL/min). The oven temperature was initially kept at 40 °C for 5 min. Then gradually increased to 250 °C at 2 °C/min rate. Held for 15 min and finally raised to 270 °C at 10 °C/min. Volatile compounds were injected at 250 °C automatically with the splitless mode. The individual peaks were analysed using the GC-MSolution package, Version 2.72. Identification of compounds was carried out using Adams [29], NIST 11, Wiley 9 and FFNSC 2 mass spectral database. 

### 2.7. Sensory Evaluations

Cheese samples were also evaluated for their sensory characteristics by a panel of 15 assessor members (nine men and six women, from 25 to 54 years old). All panelists were trained at the Department of Agricultural, Food and Forest Sciences—University of Palermo following the ISO 8589 [30] indications. The panelists were asked to score 17 descriptive attributes regarding aspect (colour and structure uniformity), smell (intensity of odour, odour of butter, odour of milk, and unpleasant odour), taste (salty, sweet, acid, bitter, and spicy), consistency (chewiness, solubility, and grittiness following mastication) and overall acceptability. The sensory evaluation of QdO cheeses were compared to that of commercial cow’s Crescenza cheese (CCCh) purchased from a retail store. Each attribute was scored using an ordinal sensory scale of intensity from 1 (low) to 9 (high) as reported by Tidona et al. [31].

### 2.8. Statistical Analyses

Microbiological, physicochemical and sensory evaluation data were subjected to one-way variance analysis (ANOVA) using XLStat software version 7.5.2 for Excel (Addinsoft, NY, USA). The Duncan procedure and Tukey’s test was applied for pairwise comparison. Statistical significance was attributed to *p* values of *p* < 0.05 and are marked with different letters.

## 3. Results 

### 3.1. Microbiological Evolution during Cheese Productions

**Figure 2:**
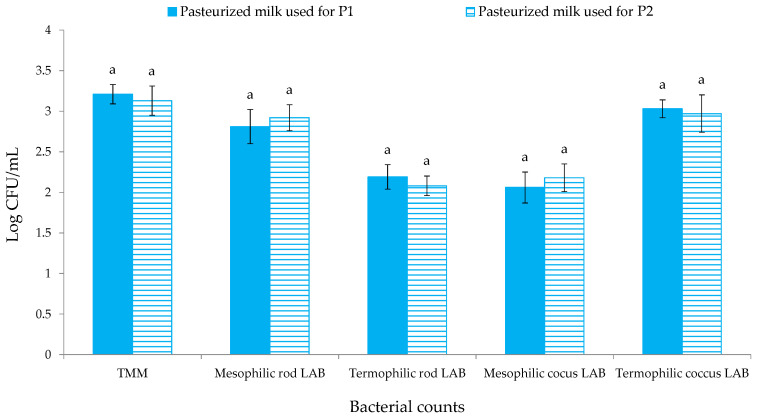
Microbial loads (Log CFU/mL) of pasteurized milk samples. Results indicate mean values and standard deviation of six determinations (carried out in duplicate for three independent productions). Abbreviations: P1, production performed with starter culture SF1 (starter formulation CR/A); P2, production performed with starter culture SF2 (starter formulation LYOBAC-D CRM); TMM, total mesophilic microorganisms; LAB, lactic acid bacteria.

The levels of the different microbial groups investigated in pasteurized milk used for QdO-P1 and QdO-P2 are reported in Figure 2. No statistically significant differences (*p* > 0.05) were observed among both pasteurized milks for the levels of TMM, mesophilic and thermophilic rod- and coccus-shaped LAB. Thermophilic coccus and mesophilic rod LAB were found at the same level (10^3^ CFU/mL) of TMM, while thermophilic rod and mesophilic coccus LAB were one log cycle lower for both matrices. The results of *Pseudomonas*, members of Enterobacteriaceae family, *L. monocytogenes*, *Salmonella* spp., *E. coli* and CPS are not reported in Figure 2 because their levels were below the detection limit for both milks. 

The bulk milks were then inoculated with the commercial *S. thermophilus* starter cultures and the levels of fermenting microorganisms in inoculated milks, curds and cheeses are reported in Table 1.

According to Duncan test, no statistically significant differences (*p* > 0.05) were found for the levels of TMM and *S. thermophilus* in all samples analysed. In particular, the level of these microorganisms for the P1 and P2 productions were almost perfectly superimposable. After inoculation with each commercial starter culture, P1 and P2 milks showed about 10^7^ CFU/mL of TMM and *S. thermophilus*. After coagulation, these microorganisms were counted at about 10^8^ CFU/g in curds for both productions showing an increase of about 1 Log cycle. Both QdO-P1and QdO-P2 cheeses reached LAB values of about 9 Log CFU/g.

### 3.2. Physicochemical Characterisation of Cheeses

#### 3.2.1. Cheese Color and Hardness

Physical measurements (colorimetric parameters and hardness) of the final cheeses are reported in Table 2. The three colour parameters (L*, a*, and b*), as well as the hardness of the cheeses were significantly influenced (*p* < 0.05) by the two starter formulations. 

#### 3.2.2. Basic Chemical Composition and Antioxidant Capacity of Cheese

The final cheeses were characterized (Table 3) by an average DM of 64.08%, mainly represented by fats (54.99%) and proteins (36.39%). Although no statistically significant differences (*p* > 0.05) were observed between QdO-P1 and QdO-P2 cheeses regarding the basic compositional analysis, TBAR_S_ values registered for QdO-P1 cheese were higher than those recorded for QdO-P2 cheese. 

### 3.3. Cheese Fatty Acids Composition

FAs composition of the experimental cheeses is shown in Table 4. Saturated fatty acids (SFAs) were consistently present in QdO cheeses (66.6 and 63.8 g/100 g for QdO-P1 and QdO-P2, respectively); among these, palmitic acid (C16:0; 24.9 and 25.7 for QdO-P1 and QdO-P2, respectively) showed a higher concentration followed by stearic acid (C18:0; 9.81 and 10.5 for QdO-P1 and QdO-P2, respectively) and myristic acid (C14:0; 11.0 and 10.6 for QdO-P1 and QdO-P2, respectively).

Among MUFA, oleic acid (OA, C18:1Δ*^c^*^9^) was the most representative as fatty acid (17.1 and 20.1 for QdO-P1 and QdO-P2, respectively). All cheeses were characterized by a high content of *trans*-vaccenic acid (VA, C18:1Δ*^t^*^11^, 3.68 and 3.91 for QdO-P1 and QdO-P2, respectively) while the *cis*-vaccenic acid (C18:1Δ*^c^*^11^) showed a much lower concentration, barely 0.64 and 0.59 for QdO-P1 and QdO-P2, respectively. Regarding PUFA, linoleic acid (LA, C18:2Δ*^c^*^9,*c*12^) was the most representative FAs (1.91 and 1.86 for QdO-P1 and QdO-P2, respectively). The use of different *S. thermophilus* strains determined significant differences in FAs composition of QdO cheeses. Considering the short-chain (SCFA; C4:0–C8:0) and medium-chain (MCFA; C10:0–C15:0) FAs, QdO-P1 cheeses showed significantly higher concentrations than QdO-P2 cheeses, except for myristoleic acid (C14:1Δ*^c^*^9^) which showed no significant differences. Significant differences were also found between long-chain FAs (LCFA; C16:0–C24:0). In particular, palmitic, stearic, oleic, *trans*-vaccenic, *γ*-linolenic, eicosenoic acid and the other C18:1 isomers, were significantly higher in QdO-P2 cheeses than in QdO-P1 cheeses. An opposite trend was registered for heptadecenoic (C17:1), *cis*-vaccenic (C18:1Δ*^c^*^11^), linoleic, *α*-linolenic (ALA, C18:3Δ*^c^*^9,*c*12,*c*15^), eicosadienoic acid (C20:2Δ*^c^*^11,*c*14^), and the other C18:2 isomers, that were significantly higher in P1. The other identified LCFAs did not show significant differences among cheese productions.

Individual fatty acid determined differences in SFA (*p* = 0.001), PUFA (*p* = 0.005), n6 PUFA (*p* = 0.013) and n3 PUFA (*p* = 0.002) which were higher in QdO-P1 rather than in QdO-P2 cheeses, while MUFA (*p* < 0.001) were at the highest concentrations in QdO-P2 cheeses. The health indices investigated were also influenced by the type of inoculum used: both n6/n3 ratio and HPI were higher in QdO-P2 cheeses (*p* = 0.002 and *p* = 0.004, respectively). 

### 3.4. Chemical Composition of Volatile Organic Compounds

The analysis of VOCs of the two QdO cheeses was carried out by SPME-GC/MS and the results are reported in Table 5. Relevant qualitative and quantitative differences were evaluated between the productions carried out with the two SFs.

Twenty-one compounds of different chemical classes (alcohols, ethers, aldehydes, ketones, carboxylic acids, esters, amines, and monoterpenes) were identified among cheeses. Ketones represented the major VOCs class of QdO-P1 cheeses (41.21 ± 0.96 ppm), followed by esters (19.32 ± 0.56 ppm) and ethereal compounds (9.03 ± 0.21 ppm). Among ketones, acetoin was the most abundant compound (31.33 ± 0.73 ppm). In descending order of quantity, the second is phenethyl hexanoate (17.25 ± 0.40 ppm). QdO-P2 cheeses showed a higher presence of short-chain carboxylic acids (acetic acid and butanoic acid) (21.25 ± 0.49 ppm), straight-chain esters (ethyl acetate, ethyl butanoate, ethyl hexanoate, ethyl octanoate, ethyl decanoate) (25.52 ± 0.60 ppm), and a lower concentration of ketones (1.49 ± 0.04 ppm) than QdO-P1 cheeses.

Aldehydes were negligible in QdO-P2 cheeses, but were detected at very low concentrations (benzaldehyde, 0.33 ± 0.01 ppm) in QdO-P1 cheeses. Significant differences were found among QdO-P1 and QdO-P2 cheeses for the production of ethyl ether (9.03 ± 0.21 and 10.33 ± 0.24 ppm for, respectively) and ethyl benzene, 1,3-dimethyl benzene and 1-butenyl benzene. Among terpenes sylvestrene was found in QdO-P2 cheeses. Among amines only dimethylamine (4.94 ± 0.39 ppm) was detected in QdO-P1 cheeses.

### 3.5. Sensory Aspects of Cheeses

**Figure 3:**
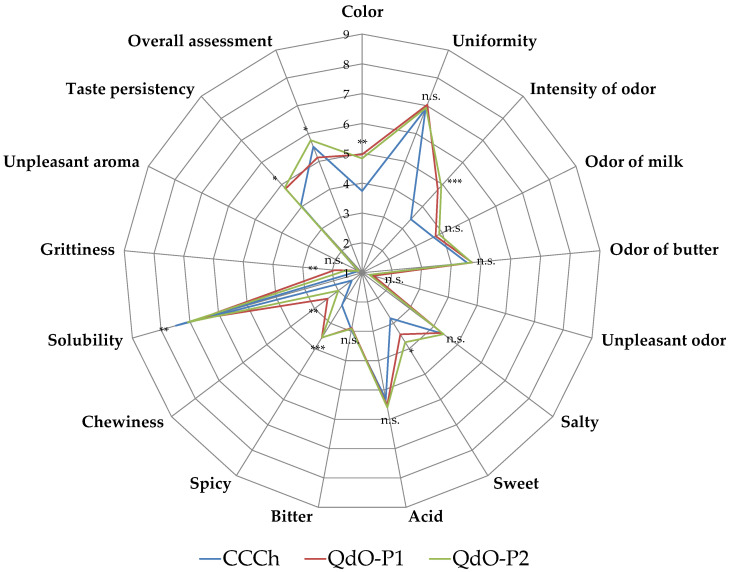
Spider diagrams of descriptive sensory analysis of cheeses. Abbreviations: CCCh, commercial crescenza cheese; QdO-P1, Quadrello di ovino cheese produced with starter culture SF1 (starter formulation CR/A); QdO-P2, Quadrello di ovino cheese produced with starter culture SF2 (starter formulation LYOBAC-D CRM). *p* value: * *p* < 0.05; ** *p* < 0.01; *** *p* < 0.001; n.s., not significant.

The sensory characteristics of QdO cheeses were evaluated by judges of different age and gender (Figure 3). In particular, the sensory characteristics of both QdO cheeses were compared to those of commercial Crescenza cheese (CCCh) taken as reference cheeses. According to the Tukey’s test, no statistically significant differences (*p* > 0.05) were found for uniformity, odour of milk, odour of butter, salty, acid, bitter and unpleased odour and aroma, while different levels of significance emerged for all other attributes evaluated.

QdO-P1 and QdO-P2 cheeses were scored at similar level by the panelists for colour, intensity of odour, sweet, spicy and taste persistency. All these attributes reached the highest in QdO. Solubility of CCCh cheese was comparable to that of QdO-P2 cheese. CCCh and QdO-P2 cheeses were not statistically different also for overall assessment, intended as the degree of overall satisfaction.

## 4. Discussion

Sicily is a southern Italian region where cheese making is a very ancient daily activity [32]. Up to date, five out of 33 traditional cheeses produced in Sicily enjoy a PDO status: Vastedda della valle del Belìce, Pecorino Siciliano, Piacentinu Ennese, Provola dei Nebrodi, and Ragusano. Among these, only ewe’s milk Vastedda della valle del Belìce cheese can be considered a fresh product [33]. Except this particularly rare example, in Italy ewe’s milk is generally transformed to produce hard or semi-hard cheeses, especially in the south part of the country [17]. 

The main hypothesis of this work was that a new typology of cheese could be produced from ewe’s milk and that this fresh cheese could be well appreciated by consumers. With this in mind, QdO cheese was processed with commercial culture preparations of *Streptococcus thermophilus*, typical thermophilic dairy LAB [34], used as starters for Crescenza cheese production [31,35,36]. Among fresh cheese, Crescenza is an Italian product particularly appreciated by consumers [37]. It is a soft rindless cheese with a very short ripening time, processed from pasteurized cow’s milk in the northern Italy, mainly in Lombardia region [38]. 

Regarding the inoculums for curd acidification, two different commercial preparations were used in order to evaluate the influence of different strains on the final characteristics of QdO cheese. The metabolic activities of *Streptococcus* strains are particularly flexible and diverse [39] and metabolomics are being widely applied for food quality analyses [40]. Thus, the use of different starter strains might provide unique metabolomics for the final products. For this reason, in this work two commercial starter preparations were used.

The pasteurised bulk milks were microbiologically investigated, and the densities of LAB were almost at the same level of TMM. These results might be ascribable to the ability of the indigenous milk bacteria to survive during the pasteurization process [41,42], but also to a post-pasteurization contamination [43,44]. Furthermore, this phenomenon seems to be common in pasteurized ewe’s milk [8,16,45]. All undesired bacteria (*Pseudomonas*, members of Enterobacteriaceae family, *L. monocytogenes*, *Salmonella* spp., *E. coli* and CPS) were undetectable. Barbaccia et al. [45] reported similar findings for pasteurized ewes’ milk used for another fresh ewe’s milk cheese production, Pecorino “Primosale”. After inoculation, starter cultures were monitored during cheese making. They increased until 10^8^ CFU/g in curds as a consequence of whey draining [46]. Both QdO cheese productions showed LAB levels of about 9 Log CFU/g following the general trend commonly observed for ovine pressed cheeses [8,47].

The cheeses were analyzed for several quality parameters, first of all for their physical characteristics (color and hardness). Color represents an important quality index of soft cheeses, including Crescenza type, that strongly affects the acceptability by consumers [48]. The three color parameters lightness, redness and yellowness of QdO cheese were, on average 87.31, −4.29, and 16.03, respectively. These values are comparable to those of the only fresh ewe’s milk cheese (Vastedda della valle del Belìce) produced in Sicily [49]. Regarding redness, the results of QdO cheeses are almost identical to that of Roncal cheese [50], while yellowness is quite lower than those characterizing several ripened cow’s milk cheeses. The initial b* values of soft cow’s milk Stracchino (synonymous of Crescenza) cheeses are in the range 11.5–12.0 [51]. Comi et al. [48] stated that an increase of yellowness in Stracchino cheese, impacts both cheese shelf life and acceptability. An increase of yellowness is commonly observed during storage of high moisture cheeses [52]. In this study, the hardness of QdO cheese, which is the force required to obtain a given deformation of the sample, was 0.22 N/mm^2^ on average. This value is extremely lower than those estimated for “Primosale” cheese for which hardness was in the range 0.98–1.10 N/mm^2^ [8]. For this reason, the texture of Crescenza type cheese is considered soft and somewhat viscous [53]. 

The basic chemical composition of QdO cheese showed an average content of fats and proteins of 35.24 and 23.32%, respectively, which are much higher than the levels reported for Crescenza cheese generally characterized by 24% of fats and 14% of proteins [31,36]. These results were quite expected, due to the high content of dry matter, proteins and fats of ewe’s milk [54]. Furthermore, the compositional analysis of ovine QdO cheeses showed data comparable to those measured for other ovine fresh cheeses such as pressed Primosale cheese and stretched Vastedda-like cheese [8,16]. QdO-P1 cheese showed a higher antioxidant activity, in terms of TBAR_S_, than QdO-P2 cheese. The lower values registered for the latter cheese are presumably due to the higher content in polyphenols of the bulk milk [55] or to the high ability of SF2 starter bacteria to inhibit lipid oxidation [56,57].

Regarding the composition of FAs, both experimental cheeses showed profiles comparable to those detected for other Sicilian ewe’s milk cheeses [49,58]. The two productions were characterized by different levels of SFA, MUFA and PUFA. Bacterial metabolism during cheese processing clearly contributed to the final cheese FAs profiles. The lipolytic activity of the two commercial strain preparations could be characterized by a different enzyme kinetics and act selectively against the FAs present in milk triglycerides, leading to a different concentration of free FAs (FFA) on which beta-oxidation occurs [59]. Furthermore, it could be possible that elongation reactions can lead to the re-elaboration of the FFA present. Barely a few studies evaluated the effect of starter cultures on cheese FAs composition, obtaining conflicting results: Gursoy et al. [60] reported an effect of different bacteria inoculums on the final FAs composition of white pickle cheese, while Branciari et al. [61] registered no effect on Pecorino cheese.

VOCs emitted from food products are decisive for the perception of aroma and taste [62]. Recently, the relationship between VOCs and sensory attributes has been successfully evaluated for Gorgonzola cheese [63]. 

QdO cheese was characterized by alcohols, ethers, aldehydes, ketones, carboxylic acids, esters, amines, and monoterpenes. The class of carboxylic acids is mostly present in cheeses [64]. In particular, a significant increase in ester and ketones can be induced by ruminants’ diet [65]. Acetoin was the most abundant ketone detected in QdO cheese. This chemical derives from the metabolism of lactose and citrate and is a common compound found in many other cheeses made with sheep and goat milk; it is responsible for the smell of butter perceived by panelists. Phenethyl hexanoate, the only ester that does not derive from linear FAs, was the second most abundant compound of this novel cheese. Carboxylic acids can be formed through lipolysis, proteolysis and fermentation of lactose. The presence of these acids provides dairy products with their characteristic aroma. Among the main acids detected, butanoic acid is characterized by a rancid and unpleasant smell [64]. According to Bontinis et al. [66], esters are originated by esterification between SCFA and ethanol, derived from the catabolism of amino acids. The presence of ethyl esters (mainly ethyl acetate, ethyl butanoate and ethyl octanoate) is characteristic of ovine cheeses. Among these compounds, ethyl butanoate, ethyl hexanoate and ethyl octanoate derive from acids and possess the typical smell of caprine cheese. Aldehydes are generally transient compounds in cheeses as they break down into acids and alcohols [67] and were detected at very low levels in QdO cheeses. Among these, benzaldehyde has a typical almond aroma, and it has been detected in other works aimed to characterize cheese flavours [68,69]. Among terpenes which derive from plants and are transferred to dairy products through animal feeding [69], only the monoterpene hydrocarbon sylvestrene was found in QdO-P2 cheeses. On the other hand, regarding amines, produced via decarboxylation of amino acids [70], only dimethylamine was detected in QdO-P1 cheeses. This amine is characterized by fruity, alcoholic, and varnish flavour notes [71]. Generally, the concentrations of amines in fresh cheeses are lower than those found in long ripened cheeses [72].

Before commercialization of novel food products, the evaluation of sensory traits plays an important role in the assessment of overall acceptability [73]. For this reason, QdO cheeses were subjected to a sensory panel evaluation and their sensory traits were compared to those of Crescenza cheese. In particular, the novel ewe’s cheese was characterized by a higher level of colour, intensity of odour, sweet, spicy and taste persistency than cow’s Crescenza cheese, no matter which starter cultures was used. These results were quite predictable since ewe’s milk determines higher taste complexity in the final transformation products than cow’s milk [74].

## 5. Conclusions

This study provided an extended analysis on the microbiological, physicochemical, VOCs profile and sensory characteristics of a novel soft ewe’s milk cheese produced at industrial scale level with two defined commercial *S. thermophilus* starters. The microbiological analysis demonstrated the suitability of *S. thermophilus* to be used as fermenting agent in QdO cheese production. Lowest secondary lipid oxidation was observed in QdO-P2 cheese and, although the differences of VOCs emitted from the two QdO cheeses were not perceived by the panelists, they showed a higher appreciation for QdO-P2 cheese that, in terms of judges’ preference was comparable to commercial Crescenza cheese. These results clearly highlighted the huge marketability perspectives of QdO cheese and provided evidence on the possibility to enlarge the dairy product portfolio of fresh ewe’s cheeses produced in Sicily.

## Figures and Tables

**Table 1:** Growth of commercial starter LAB during Quadrello di ovino cheese productions.

Samples	Bacterial Counts	
	TMM	*S. thermophilus*
Inoculated milk		
P1	6.84 ± 0.21 ^a^	7.09 ± 0.12 ^a^
P2	7.03 ± 0.13 ^a^	7.26 ± 0.16 ^a^
Curd		
P1	7.77 ± 0.23 ^a^	7.98 ± 0.18 ^a^
P2	7.92 ± 0.20 ^a^	8.02 ± 0.22 ^a^
Cheese		
QdO-P1	8.82 ± 0.11 ^a^	8.99 ± 0.29 ^a^
QdO-P2	8.73 ± 0.14 ^a^	8.89 ± 0.11 ^a^

Units are log CFU/mL for liquid samples and log CFU/g for solid samples. Results indicate mean values ± S.D. of nine plate counts (carried out in triplicate for three independent productions). Data within a column followed by the same letter are not significantly different according to Duncan test. Abbreviation: TMM, total mesophilic microorganisms; *S. Streptococcus*; P1, production performed with starter culture SF1 (starter formulation CR/A); P2, production performed with starter culture SF2 (starter formulation LYOBAC-D CRM); QdO, Quadrello di ovino cheese.

**Table 2:** Physical parameters of Quadrello di ovino cheeses.

Parameters	Samples	
	QdO-P1	QdO-P2
Lightness (L*)	88.32 ± 0.30 ^a^	86.30 ± 0.63 ^b^
Redness (a*)	−3.81 ± 0.28 ^a^	−4.76 ± 0.24 ^b^
Yellowness (b*)	15.18 ± 0.69 ^b^	16.88 ± 0.08 ^a^
Hardness, N/mm^2^	0.18 ± 0.02 ^b^	0.26 ± 0.03 ^a^

Results indicate mean values ± S.D. of nine determination (carried out in triplicate for three inde-pendent productions). Data within a line followed by the same letter are not significantly different according to Duncan test. Abbreviation: QdO-P1, Quadrello di ovino cheese produced with starter culture SF1 (starter formulation CR/A); QdO-P2, Quadrello di ovino cheese produced with starter culture SF2 (starter formulation LYOBAC-D CRM).

**Table 3:** Compositional characteristics of Quadrello di ovino cheeses.

Parameters	Samples	
	QdO-P1	QdO-P2
Dry matter (DM), %	63.17 ± 1.47 ^a^	64.99 ± 0.13 ^a^
Ash, %	5.57 ± 0.08 ^a^	5.53 ± 0.05 ^a^
Protein, %	35.52 ± 0.89 ^a^	37.26 ± 0.91 ^a^
Fat, %	54.99 ± 1.01 ^a^	54.98 ± 0.88 ^a^
NaCl, %	1.68 ± 0.08 ^a^	1.54 ± 0.01 ^a^
PI, %	1.02 ± 0.04 ^a^	1.01 ± 0.08 ^a^
POV, meq peroxide/kg fat	0.42 ± 0.01 ^a^	0.44 ± 0.03 ^a^
TBARs, MDA mg/kg DM	42.71 ± 0.74 ^a^	36.69 ± 0.37 ^b^

Results indicate mean values ± S.D. of nine determination (carried out in triplicate for three independent productions). Data within a line followed by the same letter are not significantly different according to Duncan test. Abbreviation: QdO-P1, Quadrello di ovino cheese produced with starter culture SF1 (starter formulation CR/A); QdO-P2, Quadrello di ovino cheese produced with starter culture SF2 (starter formulation LYOBAC-D CRM); PI, proteolysis index; POV, peroxide value; TBARs, thiobarbituric acid-reactive substances; MDA, malonylaldehyde.

**Table 4:** Fatty acid profile (g/100 g FA) of Quadrello di ovino cheeses.

Fatty Acids	Samples	
	QdO-P1	QdO-P2
Total FAs, % DM	48.38 ± 0.05 ^a^	49.80 ± 1.24 ^a^
C4	1.67 ± 0.04 ^a^	1.45 ± 0.00 ^b^
C6	1.88 ± 0.02 ^a^	1.44 ± 0.03 ^b^
C8	2.31 ± 0.01 ^a^	1.71 ± 0.03 ^b^
C10:0	7.25 ±0.02 ^a^	5.50 ± 0.06 ^b^
C10:1Δ*^c^*^9^	0.28 ± 0.00 ^a^	0.22 ± 0.00 ^b^
C12:0	4.11 ± 0.02 ^a^	3.43 ± 0.02 ^b^
C12:1Δ*^c^*^5^	0.13 ± 0.00 ^a^	0.11 ± 0.00 ^b^
C14:0	11.01 ± 0.05 ^a^	10.67 ± 0.10 ^b^
C14:1Δ*^c^*^9^	0.23 ± 0.00 ^a^	0.23 ± 0.00 ^a^
C15:0	1.89 ± 0.01 ^a^	1.77 ± 0.02 ^b^
C15:1Δ*^c^*^14^	0.51 ± 0.00 ^a^	0.48 ± 0.00 ^b^
C16:0	24.84 ± 0.05 ^b^	25.73 ± 0.09 ^a^
C16:1Δ*^c^*^9^	0.23 ± 0.00 ^a^	0.22 ± 0.00 ^a^
C17:0	1.47 ± 0.00 ^a^	1.46 ± 0.01 ^a^
C17:1Δ*^c^*^9^	0.29 ± 0.00 ^a^	0.24 ± 0.00 ^b^
C18:0	9.81 ± 0.09 ^b^	10.5 ± 0.04 ^a^
C18:1Δ*^c^*^9^ (OA)	17.11 ± 0.04 ^b^	20.14 ± 0.07 ^a^
C18:1Δ*^c^*^11^	0.64 ± 0.00 ^a^	0.59 ± 0.01 ^b^
C18:1Δ*^t^*^11^	3.68 ± 0.02 ^b^	3.91 ± 0.04 ^a^
C18:1Δ*^c^*^13^	3.28 ± 0.02 ^b^	3.82 ± 0.02 ^a^
C18:2Δ*^t^*^9,*c*12^	0.88 ± 0.01 ^a^	0.62 ± 0.01 ^b^
C18:2Δ*^c^*^9,*c*12^ (LA)	1.91 ± 0.01 ^a^	1.86 ± 0.01 ^b^
C18:2Δ*^c^*^9,*t*11^ (RA)	1.20 ± 0.03 ^a^	1.30 ± 0.04 ^a^
C18:2Δ*^t^*^10,*c*12^	0.46 ± 0.00 ^a^	0.39 ± 0.01 ^b^
C18:3Δ*^c^*^9,*c*12,*c*15^ (ALA)	1.54 ± 0.00 ^a^	1.15 ± 0.00 ^b^
C18:3Δ^c6,*c*9,*c*12^ (GLA)	0.34 ± 0.00 ^b^	0.37 ± 0.00 ^a^
C20:0	0.10 ± 0.01 ^a^	0.09 ± 0.01 ^a^
C20:1Δ*^c^*^11^	0.07 ± 0.00 ^b^	0.09 ± 0.00 ^a^
C20:2Δ*^c^*^11,*c*14^	0.10 ± 0.00 ^a^	0.08 ± 0.00 ^b^
C20:3Δ*^c^*^11,*c*14,*c*17^	0.02 ± 0.00 ^a^	0.01 ± 0.00 ^a^
C20:3Δ*^c^*^8,*c*11,*c*14^	0.02 ± 0.00 ^a^	0.02 ± 0.00 ^a^
C20:4Δ*^c^*^5,*c*8,*c*11,*c*14^ (AA)	0.11 ± 0.01 ^a^	0.11 ± 0.00 ^a^
C20:5Δ*^c^*^5,*c*8,*c*11,*c*14,*c*17^ (EPA)	0.15 ± 0.02 ^a^	0.15 ± 0.00 ^a^
C22:0	0.12 ± 0.00 ^a^	0.13 ± 0.00 ^a^
C22:2Δ*^c^*^13,*c*16^	0.15 ± 0.00 ^a^	0.15 ± 0.00 ^a^
C22:4Δ*^c^*^8,*c*11,*c*14,*c*17^	0.03 ± 0.00 ^a^	0.04 ± 0.00 ^a^
C22:5Δ*^c^*^7,*c*10,*c*13,*c*16,*c*19^ (DPA)	0.12 ± 0.00 ^a^	0.10 ± 0.00 ^b^
C22:6Δ*^c^*^4,*c*7,*c*10,*c*13,*c*16,*c*19^	0.05 ± 0.00 ^a^	0.03 ± 0.00 ^b^
C24:0	0.02 ± 0.01 ^a^	0.02 ± 0.00 ^a^
SFA	66.59 ± 0.04 ^a^	63.82 ± 0.09 ^b^
MUFA	26.51 ± 0.03 ^b^	29.92 ± 0.10 ^a^
PUFA	6.94 ± 0.07 ^a^	6.25 ± 0.01 ^b^
n6	2.53 ± 0.00 ^a^	2.49 ± 0.01 ^b^
n3	1.86 ± 0.03 ^a^	1.45 ± 0.01 ^b^
n6/n3	1.36 ± 0.02 ^b^	1.71 ± 0.00 ^a^
HPI	0.42 ± 0.00 ^b^	0.47 ± 0.00 ^a^

Results indicate mean values ± S.D. of nine determination (carried out in triplicate for three independent productions). Data within a line followed by the same letter are not significantly different according to Duncan test. Abbreviation: QdO-P1, Quadrello di ovino cheese produced with starter culture SF1 (starter formulation CR/A); QdO-P2, Quadrello di ovino cheese produced with starter culture SF2 (starter formulation LYOBAC-D CRM); FA = fatty acid; OA = oleic acid; DM = dry matter; LA = linoleic acid; ALA = *α*-linolenic acid; GLA = *γ*-linolenic acid; RA = rumenic acid; AA = arachidonic acid; EPA = eicosapentaenoic acid; DPA = docosapentaenoic acid; SFA = saturated fatty acids; MUFA = monounsaturated fatty acids; PUFA = polyunsaturated fatty acids; HPI = health promoting index.

**Table 5:** Abundance of VOCs emitted from Quadrello di ovino cheeses.

Retention Time (min.sec)	Compounds ^a^ (Common Names)	Samples		Identification
		QdO-P1	QdO-P2	
	Σ Alcohols	4.30 ± 0.34 ^a^	1.49 ± 0.03 ^b^	
6.30	3-Methyl-1-butanol	4.30 ± 0.34 ^a^	1.49 ± 0.03 ^b^	b
	Σ Ethers	9.03 ± 0.21 ^b^	10.33 ± 0.24 ^a^	
2.75	Ethyl ether	9.03 ± 0.21 ^b^	10.33 ± 0.24 ^a^	b, c
	Σ Aldehydes	0.33 ± 0.01 ^a^	n.d. ^b^	
19.45	Benzaldehyde	0.33 ± 0.01 ^a^	n.d. ^b^	b, c
	Σ Ketones	41.21 ± 0.96 ^a^	1.49 ± 0.04 ^b^	
4.10	2-Pentanone	5.93 ± 0.14 ^a^	n.d. ^b^	b
5.40	Butan-2-one-3-hydroxy (Acetoin)	31.33 ± 0.73 ^a^	n.d. ^b^	b
8.50	2-Hexanone	1.22 ± 0.03 ^a^	n.d. ^b^	b
15.35	2-Heptanone	n.d. ^b^	1.09 ± 0.03 ^a^	b
29.45	2-Nonanone	2.73 ± 0.06 ^a^	0.40 ± 0.01 ^b^	b
	Σ Carboxylic Acids	1.29 ± 0.10 ^b^	21.25 ± 0.49 ^a^	
4.40	Acetic acid	1.29 ± 0.10 ^b^	15.35 ± 0.35 ^a^	b, c
9.45	Butanoic acid	n.d. ^b^	5.90 ± 0.14 ^a^	b
	Σ Esters	19.32 ± 0.56 ^b^	25.52 ± 0.60 ^a^	
4.27	Ethyl acetate	n.d. ^b^	8.43 ± 0.20 ^a^	b, c
9.56	Ethyl butanoate	n.d. ^b^	9.83 ± 0.23 ^a^	b
14.45	Phenethyl hexanoate (Phenethyl caproate)	17.25 ± 0.40 ^a^	n.d. ^b^	b
22.55	Ethyl hexanoate (Ethyl caproate)	0.23 ± 0.02 ^b^	3.40 ± 0.08 ^a^	b
37.34	Ethyl octanoate (Ethyl caprylate)	1.14 ± 0.09 ^b^	2.33 ± 0.05 ^a^	b, c
50.94	Ethyl decanoate (Ethyl caprate)	0.70 ± 0.05 ^b^	1.53 ± 0.04 ^a^	b
	Σ Amines	4.94 ± 0.39 ^a^	n.d.	
2.55	Dimethylamine	4.94 ± 0.39 ^a^	n.d.	b
	Σ Monoterpenes	n.d. ^b^	0.20 ± 0.00 ^a^	
24.60	Sylvestrene	n.d. ^b^	0.20 ± 0.00 ^a^	b
	Σ Others	1.11 ± 0.03 ^b^	1.45 ± 0.04 ^a^	
12.40	Ethyl benzene	0.28 ± 0.01 ^b^	0.67 ± 0.02 ^a^	b
13.00	1,3-Dimethyl benzene	0.83 ± 0.02 ^a^	0.39 ± 0.01 ^b^	b
30.01	1-Butenyl benzene	n.d. ^b^	0.39 ± 0.01 ^a^	b

Results are expressed in parts per million (ppm). ^a^ Compounds are tabulated according to the organic class and to retention time; b comparison with mass spectrum libraries; c co-elution with authentic sample. Data within a line followed by the same letter are not significantly different according to Duncan test. Abbreviation: QdO-P1, Quadrello di ovino cheese produced with starter culture SF1 (starter formulation CR/A); QdO-P2, Quadrello di ovino cheese produced with starter culture SF2 (starter formulation LYOBAC-D CRM); n.d., not detectable.

## Data Availability

All data included in this study are available upon request by contacting the corresponding author.
